# Impaired DNA-binding affinity of novel *PAX6* mutations

**DOI:** 10.1038/s41598-020-60017-2

**Published:** 2020-02-20

**Authors:** Seowhang Lee, Seung-Han Lee, Hwan Heo, Eun Hye Oh, Jin-Hong Shin, Hyang-Sook Kim, Jae-Ho Jung, Seo Young Choi, Kwang-Dong Choi, Hakbong Lee, Changwook Lee, Jae-Hwan Choi

**Affiliations:** 1 Department of Biological Sciences, School of Life Sciences, Ulsan National Institute of Sciences and Technology, Ulsan, South Korea; 2 Department of Neurology, Chonnam National University Medical School, Gwangju, South Korea; 3 Department of Ophthalmology, Chonnam National University Medical School, Gwangju, South Korea; 4 Department of Neurology, Pusan National University School of Medicine, Research Institute for Convergence of Biomedical Science and Technology, Pusan National University Yangsan Hospital, Yangsan, South Korea; 5 Department of Ophthalmology, Seoul National University Hospital, Seoul, South Korea; 6 Department of Neurology, Pusan National University Hospital, Pusan National University School of Medicine and Biomedical Research Institute, Busan, Korea

**Keywords:** Genetic predisposition to disease, Hereditary eye disease

## Abstract

Mutations in human *PAX6* gene are associated with various congenital eye malformations including aniridia, foveal hypoplasia, and congenital nystagmus. These various phenotypes may depend on the mutation spectrums that can affect DNA-binding affinity, although this hypothesis is debatable. We screened *PAX6* mutations in two unrelated patients with congenital nystagmus, and measured DNA-binding affinity through isothermal titration calorimetry (ITC). To elucidate phenotypic differences according to DNA-binding affinity, we also compared DNA-binding affinity among the previously reported *PAX6* missense mutations within the linker region between two subdomains of the paired domain (PD). We identified two novel mutations of *PAX6* gene: c.214 G > T (p.Gly72Cys) and c.249_250delinsCGC (p.Val84Alafs*8). Both were located within the linker region between the two subdomains of the PD. ITC measurement revealed that the mutation p.Val84Alafs*8 had no DNA-binding affinity, while the p.Gly72Cys mutation showed a decreased binding affinity (Kd = 0.58 μM) by approximately 1.4 times compared to the wild type-PAX6 (Kd = 0.41 μM). We also found that there was no close relationship between DNA-binding affinity and phenotypic differences. Our results suggest that the DNA-binding affinity alone might be insufficient to determine *PAX6*-related phenotypes, and that other modifier genes or environmental factors might affect phenotypes of the *PAX6* gene.

## Introduction

The *PAX6* gene (MIM 607108) is a highly conserved gene to play an essential role in eye development^1,2^. It was identified as a candidate gene for congenital aniridia by positional cloning, and acts as a “master control” gene for normal oculogenesis by encoding a transcriptional regulatory protein^2,3^. The protein has two DNA-binding domains, a paired domain (PD) and a homeodomain, that recognize different DNA-binding sequences of target genes^4–8^.

To date, more than 400 heterozygous mutations within the *PAX6* gene have been identified in the Human *PAX6* Allelic Variant Database^2,9–11^. Classically, *PAX6* mutation is associated with an aniridia, but may also causes a variety of non-aniridia phenotypes such as microcornea, foveal hypoplasia, keratitis, and optic nerve malformations^12–19^. According to a comprehensive review of *PAX6* mutations, phenotypes have relied mostly on mutation spectrums and distributions; aniridia is typically caused by truncating mutations, while non-aniridia is generally caused by missense mutations^2,11,20^. This has been explained by differences in the DNA-binding affinity and transcriptional activation, but this hypothesis is debatable^21–24^.

We report two novel *PAX6* mutations associated with infantile nystagmus syndrome (INS). We also analyzed the DNA-binding affinity of mutant *PAX6* to elucidate the relationship between DNA-binding affinity and phenotypic differences.

## Results

### Clinical characteristics

**Figure 1:**
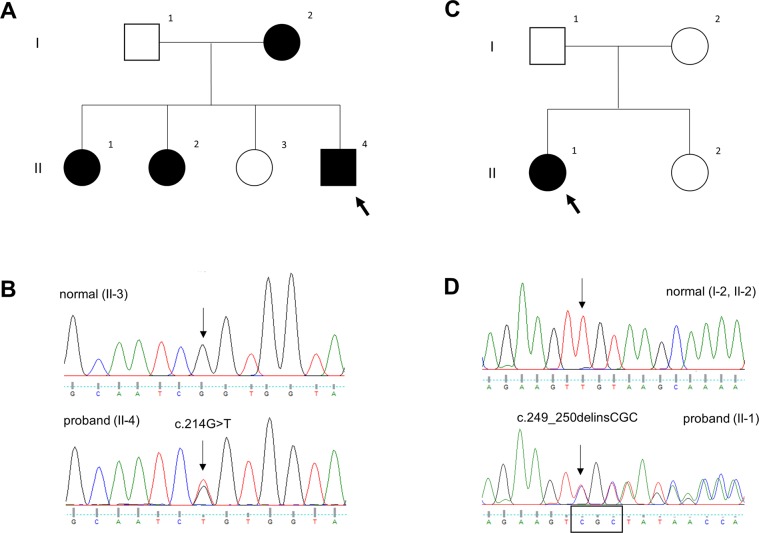
Pedigree of the patients and sequencing results of *PAX6* gene. Pedigree (**A**) and sequencing result (**B**) of case 1. Solid symbols (squares = males, circles = females) indicate clinically affected individuals; and open symbols, unaffected individuals. The proband (II-4) is indicated by an arrow. DNA sequences for an unaffected member (II-3, upper) and the proband (II-4, bottom). The chromatograms of *PAX6* genomic sequences reveals a heterozygous missense mutation c.214 G > T of exon 6 in the proband. This is predicted to result in the substitution of the highly conserved glycine by cysteine at position 72. Pedigree (**C**) and sequencing result (**D**) of case 2. The proband (II-1) is indicated by an arrow. In the proband, the chromatograms shows a deletion of nucleotides c.249 to c.250 (TG), replaced by nucleotides CGC in exon 6. This mutation causes a premature termination codon 8 amino acids downstream.

**Figure 2:**
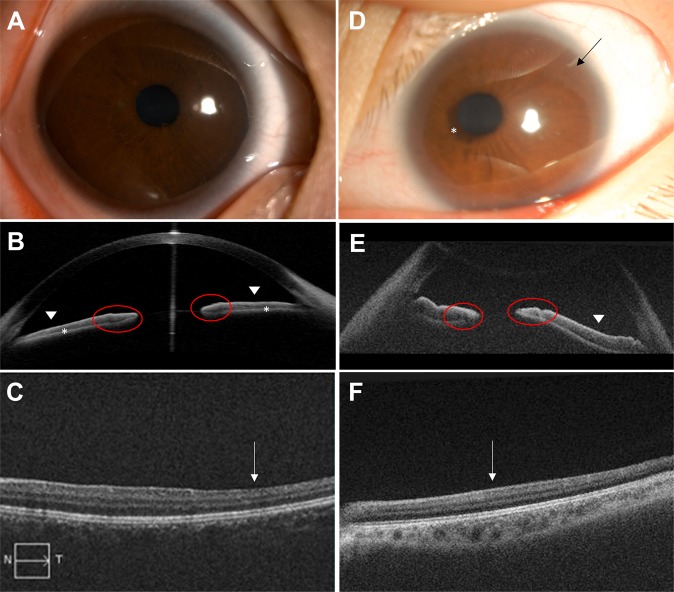
Clinical characteristics of the patients. (**A–C**) In case 1, anterior segment photography of the right eye (**A**) shows no obvious iris anomaly except for minimal nasally displaced pupil. Anterior segment optical coherence tomography (AS-OCT) (**B**) reveals a generalized iris stromal hypoplasia (white asterisk) with a lack of contraction furrows and crypts on the iris ciliary zone (white arrow head) and flattening of collarette pupillary zone (red circle). Posterior segment OCT (PS-OCT) (**C**) shows the absence of foveal pit (white arrow). (**D–F**) In case 2, anterior segment photography of the left eye (**D**) reveals nasally displaced pupil with iris ectropion uvea (white asterisk) and peripheral corneal dystrophy (black arrow). AS-OCT (**E**) shows a lack of contraction furrows and crypts on the iris ciliary zone (white arrow head) and flattening of collarette pupillary zone (red circle). There is the absence of foveal pit in PS-OCT (**F**, white arrow).

Detailed ophthalmic and oculomotor findings are described in Table 1. Case 1 (P1-MSH, II-4, Fig. 1A) was a 5-year-old male with poor visual acuity and congenital nystagmus. Slit lamp examination with anterior segment photography showed a corectopia with normal-appearing iris and no transillumination (Fig. 2A), however anterior segment optical coherence tomography (AS-OCT) revealed a generalized iris stromal hypoplasia (Fig. 2B). In addition, there was a lack of contraction furrows and crypts on the iris ciliary zone and flattening of collarette pupillary zone. Posterior segment OCT (PS-OCT) showed foveal hypoplasia of grade 4 (Fig. 2C). His mother and two sisters also showed the same clinical features with additional phenotypical characteristics including presenile cataracts in two (I-2 and II-1), exotropia in two (I-2 and II-2), and optic nerve head hypoplasia in one (II-2). Case 2 (P2-AJY, II-1, Fig. 1C) was a 14-year-old female with poor visual acuity, congenital nystagmus and exotropia. She also had a peripheral corneal dystrophy and corectopia without transillumination on slit lamp examination (Fig. 2D). AS-OCT demonstrated iris anomalies including a lack of contraction furrows and crypts on the iris ciliary zone and flattening of the collarette pupillary zone (Fig. 2E). Foveal hypoplasia of grade 4 was observed in PS-OCT (Fig. 2F). Her mother and younger sister were clinically normal for both eyes.

**Table 1:** Clinical and genetic characteristics of two families with *PAX6* mutation.

Patient ID	Sex/Age	Mutation	Exon (Domain)	Protein change	BCVA (OD/OS)	Iris anomaly	Strabismus	Nystagmus	OCT	Other features
P1-MSH										
I-2	F/42	c.214 G > T	Ex 6 (PD)	p.Gly72Cys	0.3/0.3	Iris hypoplasia	XT	HJ (PAN)	FH (G4)	cataract, corectopia
II-1	F/17				0.2/0.3	Iris hypoplasia	(—)	HJ (PAN)	FH (G4)	cataract, corectopia
II-2	F/15				0.2/0.3	Iris hypoplasia	XT	HJ (PAN)	FH (G4), ONH	corectopia
II-4	M/5				0.2/0.2	Iris hypoplasia	(—)	HJ	FH (G4)	corectopia
P2-AJY										
II-1	F/14	c.249_250delinsCGC	Ex 6 (PD)	p.Val84Alafs*8	0.1/3m0.1	Iris hypoplasia	XT	HJ	FH (G4)	corectopia, corneal dystrophy

BCVA = best-corrected visual acuity; Ex = exon; F = female; FH = foveal hypoplasia; G4 = grade 4; HJ = horizontal jerky; M = male; OCT = optical coherence tomography; ONH = optic nerve head hypoplasia; PD = paired-box domain; PAN = periodic alternating nystagmus; XT = exotropia.

### Mutation analysis

After variant filtering, annotation, and interpretation, we identified novel mutations of *PAX6* (NM_001258462.1) in each of the two patients. These two mutations were absent in 150 normal controls and all public databases of dbSNP, ExAC, 1000 Genomes Project, and KRGDB. Three rare variants were detected in other INS-associated genes (c.8192 T > C and c.5825 G > A in *ALMS1*; c.5593 C > T in *ABCA4*), but they were unlikely to be pathogenic based on a heterozygous mutation, in silico prediction, the public database, and the clinical phenotype (see Supplementary Tables S1 and S2).

Case 1 had a heterozygous missense mutation c.214 G > T (p.Gly72Cys) in exon 6, which was predicted to result in substitution of the highly conserved glycine by cysteine at position 72 within the linker region between two subdomains of the PD (Fig. 1B). The mutation showed deleterious effect by all prediction tools, and co-segregated with disease phenotype in all affected family members (I-2, II-1 and II-2).

Case 2 had a heterozygous mutation c.249_250delinsCGC (p.Val84Alafs*8) in exon 6, resulting in a change of valine into alanine at position 84 and subsequently leading to a premature termination codon 8 amino acids downstream in the C-terminal subdomain of the PD (Fig. 1D). This mutation was not detected in unaffected family members (I-2 and II-2).

### DNA-binding affinities of *PAX6* mutations

DNA-binding affinities of the *PAX6* mutations are summarized in Table 2.

**Table 2:** DNA (P6B) binding affinities of *PAX6* mutations.

Mutation	Iris anomaly	Kd (μM)	N	△H (kJ/mol)	T△S (kJ/mol)	reference
WT	WT	0.411 ± 0.068	0.972 ± 0.012	−187 ± 4.61	−150	(—)
p.Gly72Cys	iris hypoplasia	0.579 ± 0.370	0.762 ± 0.044	−125 ± 13.2	−89.5	this study
p.Val84Alafs*8	iris hypoplasia	no binding	—	—	—	this study
p.Gly64Val	normal iris	2.520 ± 1.020	0.751 ± 0.043	−195 ± 24.2	−163	^23^
p.Gly72Ser	iris hypoplasia	1.200 ± 0.130	0.728 ± 0.009	−115 ± 3.24	−80.8	^25^
p.Gly73Asp	aniridia	0.489 ± 0.128	0.590 ± 0.014	−83.3 ± 3.59	−47.3	^26^
p.Ser74Gly	normal iris	0.699 ± 0.180	0.831 ± 0.019	−106 ± 5.18	−70.8	^27^
p.Pro76Arg	normal iris	0.740 ± 0.093	0.712 ± 0.008	−111 ± 2.82	−75.8	^24^

Kd is dissociation constant in equilibrium state; N, stoichiometry indicates the ratio of ligand-to-macromolecule binding; ΔH, enthalpy is indication of changes in hydrogen and van der Waals bonding; TΔS, entropy is indication of changes in hydrophobic interaction and conformational changes.

**Figure 3:**
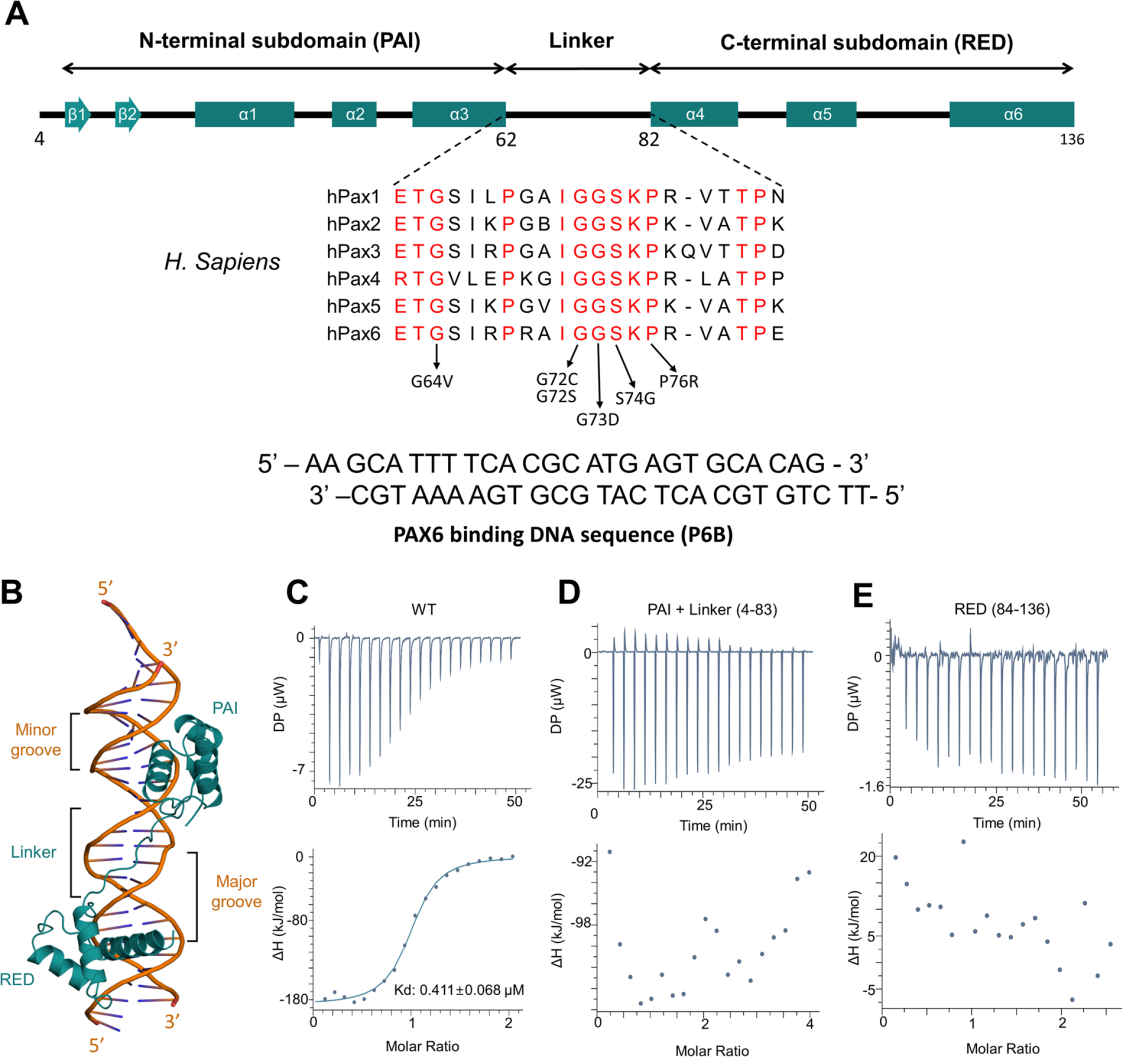
Structure and DNA binding affinity of the paired domain of PAX6. (**A**) Schematic diagram showing a primary structure of the paired domain (PD) of PAX6. The PD of PAX6 comprises N-terminal PAI and C-terminal RED subdomains, and a linker between the two subdomains. Sequences corresponding to the linker among the PAX paralogue proteins, PAX1 to 6 are aligned to highlight their conservation. Missense mutations found by previous and this study are labeled with black arrows. DNA duplex sequence (P6B) used for ITC experiments is shown below. (**B**) The structure of PAX6 (green) bound to DNA (orange) (PDB ID: 6PAX). (**C**) ITC measurement between wild-type PAX6 PD (reside: 4–136) and its target DNA (See methods for details). (**D**,**E**) ITC measurement of truncated PAX6 (**D**; residues 4–83, **E**; residues 84–136). The results show that each PAI subdomain with linker or RED subdomain alone does not bind to the DNA in solution. The upper panels shows the raw ITC data for injection of target DNA into the sample cell containing PAX6. The reaction heat of DNA and protein binding was represented as differential power (DP) between the reference and sample cells to maintain a same temperature. Molar ratio calculated by DNA: protein binding concentration and integrated as shown in the bottom panels. Experimental data indicated with solid dots and fitted in a binding curve using a one-site binding model.

Based on the crystal structure of PAX6 bound to DNA, the PD adopts two helix-turn-helix motifs (PAI and RED subdomain) that are connected by an extended loop comprising residues 62–83 (Fig. 3A)^21^. Each subdomain binds to the distinctive major groove of DNA in a bipartite manner, whereas the linker loop binds to the minor groove of the DNA and its flexibility assists the PD to accommodate appropriately to major grooves of its target DNA (Fig. 3B). To examine if these identified mutations might affect DNA binding, we measured the binding affinity between PAX6 and its target DNA using an ITC. For this experiment, DNA fragment (Pax6 binding sequence, referred to as “P6B”, see Supplementary Fig. S1) used for crystallization was prepared as described previously^21^. As shown in Fig. 3C, wild type PAX6 (WT-PAX6) could bind to the P6B with an affinity of around 0.41 μM dissociation constant (Kd) value. Interestingly, each PAI subdomain with linker or RED subdomain alone did not bind to the DNA in solution, suggesting that both subdomains might be essentially required to interact with the DNA (Fig. 3D,E). The result of PAI subdomain with linker showed that the mutation p.Val84Alafs*8 of case 2 had no DNA binding affinity (Fig. 3D).

**Figure 4:**
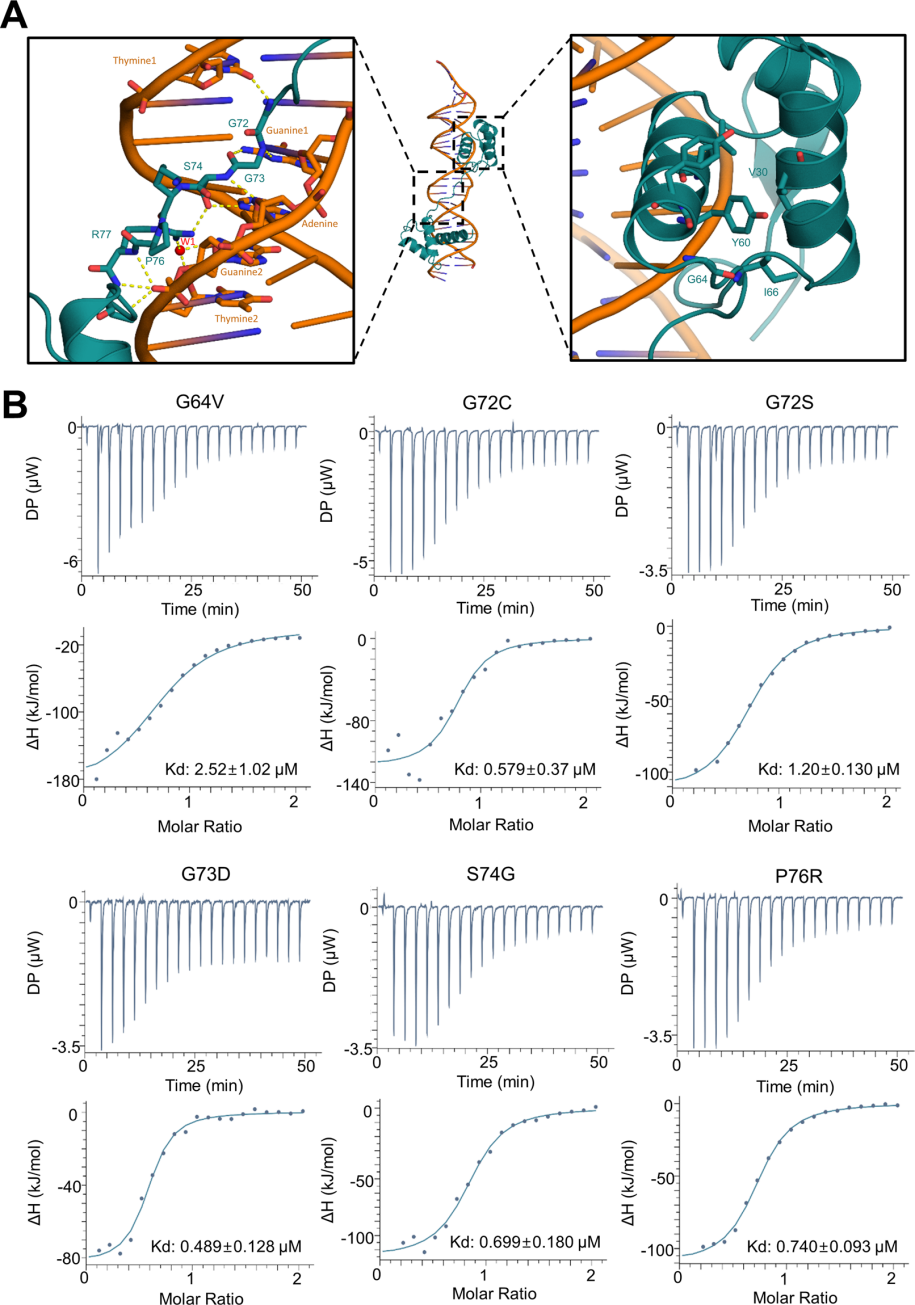
DNA binding affinity for *PAX6* missense mutations within the linker region between the two subdomains of the PD. (**A**) Close-up views show the interactions between PAX6 and its target DNA. Color scheme is the same as Fig. 3B. Oxygen and nitrogen atoms are colored red and blue, respectively. The yellow dotted lines indicate intermolecular hydrogen bonds between PAX6 and DNA. (**B**) ITC measurements of PAX6 missense mutations for DNA. Although all missense mutations show the impaired DNA-binding affinity compared to the wild-type PAX6, some (G72C, G73D, and S74G) have no dramatic effect for the DNA-binding affinity.

Next, we analyzed p.Gly72Cys (case 1 in this study), p.Gly72Ser^25^ and p.Gly73Asp^26^ mutations associated with iris anomaly within the linker region between the two subdomains of the PD. According to the crystal structure, p.Gly72 located in the center of linker could support the flexibility in this linker region (Fig. 4A). Nevertheless, the p.Gly72Cys mutant identified in this study could bind to the P6B with an affinity of 0.58 μM, suggesting that the DNA binding was mildly impaired in p.Gly72Cys mutation compared to that of WT-PAX6 (Fig. 4B). And two successive glycine residues (p.Gly72 and p.Gly73) located in the center of linker could support the flexibility in this linker region. Thus, the p.Gly72Ser mutation showed a decreased binding affinity by 2.8 times (Kd = 1.2 μM) compared to WT-PAX6, and had more meaningful effect than the p.Gly72Cys mutation (Table 2, Fig. 4B). We assumed that even if the mutation might somehow restrict the flexibility of the linker, p.Gly73 could still offer flexibility to the linker. Therefore, the linker could maintain a flexibility and retain its ability to interact with DNA. With regard to the pGly73Asp mutation, a previous study using EMSA has revealed that this mutation leads to complete loss of its binding ability for CD19 A-ins DNA sequences (see Supplementary Fig. S1) known as another target sequence of PAX6^26^. Indeed, the binding affinity of p.Gly73Asp mutation for CD19 A-ins was undetectable in our ITC measurement either (see Supplementary Fig. S2 and Table S3). Whereas, the binding affinity of the mutant for the P6B (Kd = 0.489 μM, Fig. 4B) or P6CON (Kd = 0.961 μM, see Supplementary Fig. S2 and Table S3) was unaffected compared to the WT.

Finally, we measured DNA-binding affinities of missense mutations (p.Gly64Val^23^, p.Ser74Gly^27^, p.Pro76Arg^24^) associated with non-aniridia phenotype within the linker region between the two subdomains of the PD. Previous *in silico* modeling data have suggested that the p.Ser74Gly mutation can result in the absence of side chain of the residue, and therefore might perturb the DNA binding properties of PAX6^27^. However, p.Ser74Gly and p.Pro76Arg mutations mildly impaired DNA binding (p.Ser74Gly: Kd = 0.70 μM; p.Pro76Arg: Kd = 0.74 μM) although the side chain of p.Ser74 and p.Pro76 residue made an H-bond and hydrophobic contact with deoxyribose from the minor groove, respectively (Table 2, Fig. 4B)^21^. The p.Gly64 residue was not directly involved in the DNA structure by crystal structure (Fig. 4A). However, it formed a van der Waals stack with p.Tyr60 known to play an important role in adopting a three-helix bundle structure in PAI subdomain^21^. Therefore, the change of glycine into valine might cause steric hindrance in this region, thus affecting the stability of PAX6 and eventually hindering DNA binding. Consistent with this hypothesis, the p.Gly64Val mutation showed a 6-fold decreased binding affinity compared to WT-PAX6 (Table 2, Fig. 4B). Unlike our data, a previous study using EMSA has shown that p.Gly64Val mutation exhibited an enhanced binding affinity to P6CON, a promoter sequence specifically recognized by PAX6^28^. To analyze if the mutation could exhibit different effects depending on distinct target DNA promoters, we also measured binding affinity of p.Gly64Val mutation for both P6CON and CD19A-ins DNA sequences (see Supplementary Fig. S1) known as target sequences of PAX6^28,29^. Consistent with our data, the p.Gly64Val mutation showed a decreased binding affinity for both P6CON and CD19 A-ins by 1.4 times and 2.3 times, respectively (see Supplementary Fig. S2 and Tables S3 and S4).

## Discussion

We identified two novel mutations within the *PAX6* gene in patients with iris hypoplasia and INS. One was a missense mutation (c.214 G > T, p.Gly72Cys) and the other was a truncating mutation (c.249_250delinsCGC, p.Val84Alafs*8). Both were located within the linker region between two subdomains of the PD, leading to impaired DNA-binding affinity as demonstrated by ITC measurement.

The *PAX6* gene maps to chromosome 11p13 and contains 14 exons including an alternatively spliced exon 5a^2^. It encodes a transcriptional regulator which plays an important role in oculogenesis and other developmental processes^4–8^. The PAX6 protein consists of the PD, the homeodomain, and the proline/serine/threonine-rich transactivation domain^8^. The PD is composed of two distinct DNA-binding subdomains: the N-terminal subdomain (PAI) and the C-terminal subdomain (RED) (Fig. 3A). Since the PD and homeodomain recognize different DNA-binding sequences, the PAX6 protein can have the diversity in DNA sequence recognition pattern of a variety of target genes^21,27^. In humans, *PAX6* mutations are associated with a variety of congenital eye malformations^2,11–20^. The classic phenotype is aniridia characterized by congenital absence of the iris. It is often accompanied by congenital anomalies of the cornea, lens, retina and optic nerve^12–15^. *PAX6* mutations also cause various non-aniridia phenotypes without iris abnormalities such as microcornea, foveal hypoplasia, keratitis, and optic nerve malformations^16–19^. The aniridia phenotype is predominantly associated with truncating mutations that result in a premature termination codon (PTC), while missense mutations usually lead to non-aniridia phenotypes^2,11,20^. Since missense mutations causing non-aniridia phenotypes are mostly located in the PD, partially impaired DNA-binding affinity has been proposed as a major mechanism of non-aniridia phenotypes^21–24^. Indeed, previous functional assays have demonstrated that the p.Ile87Arg mutation with no DNA-binding affinity can result in complete aniridia, while the p.Arg26Gly mutation with the relatively less impaired DNA-binding affinity can lead to a non-aniridia phenotype by a hypomorphic allele^22^.

However, this genotype-phenotype correlation of *PAX6* mutations remains controversial. Some truncating mutations have been associated with the non-aniridia phenotypes such as autosomal dominant keratitis, optic nerve hypoplasia (ONH), and congenital cataract^16,18,30^. On the contrary, total aniridia has been reported in missense mutations^22,31,32^. Sometimes, the same mutation can result in various phenotypes ranging from normal iris to complete absence of the iris among the same family members^19,31^. In our study, patients showed similar phenotypes including iris hypoplasia, INS, and foveal hypoplasia regardless of the mutation spectrum. In particular, the iris had an almost normal appearance when viewed through slit lamp biomicroscopy, but abnormal changes in the iris architecture could be detected by AS-OCT. This may imply that AS-OCT is the best tool to identify iris abnormalities associated with *PAX6* mutations, and caution must be taken when drawing conclusions about a non-aniridia phenotype without using AS-OCT^33^.

In addition, ITC measurement in our study did not show any relationship between DNA-binding affinity and phenotypic differences. DNA-binding affinities of previously reported missense mutations (p.Gly64Val^23^, p.Ser74Gly^27^, p.Pro76Arg^24^) with normal iris phenotype were more impaired than those of p.Gly72Cys (case 1 in our study) and p.Gly73Asp^26^, both being associated with iris anomalies. The p.Gly64Val mutation associated with non-aniridia phenotype showed the most impaired DNA-binding affinity. A family with p.Ser74Gly mutation had additional intellectual disabilities and epilepsy despite mildly impaired DNA-binding affinity^27^. The truncating mutation of case 2 showed a failure of DNA-binding in solution, although the patient had a full iris with abnormal architecture, rather than total absence of the iris. These observations suggest that the DNA-binding affinity alone is not sufficient to determine *PAX6*-related phenotypes, and other modifier genes or environmental factors might affect these phenotypes^31^. Various co-activators such as Sox2 and Maf, and co-repressors such as Smad3 and Nkx6.1 might play important roles in the expression of downstream targets of PAX6^34–36^. One report has revealed a suppression failure of the *PAX2* promoter activity as well as impairment of the transcriptional activation potential of *PAX6* in a patient with bilateral ONH caused by a non-sense mutation^18^. The authors speculated that *PAX2* and *PAX6* might cooperate in optic-nerve formation and that a failure of the *PAX6*-*PAX2* regulatory circuit might affect phenotypic manifestations. In addition, a recent study has revealed that PAX6 nuclear localization signal (NLS) sequence exists in the PD, especially residues 48 to 83, including residues studied in the present study^37^. Therefore, future study is needed to address whether mutations within this region could prevent PAX6 from translocating into the nucleus, resulting in phenotypic differences.

In this study, we measured the binding affinity between PAX6 and its various target DNA fragments using ITC, and confirmed that PAX6 had a comparable binding affinity for the DNA. ITC is a physical technique used to determine thermodynamic parameters of interactions in solution, and widely used to study DNA-binding affinity^38^. ITC measurement could produce quite accurate binding constants that can be used to exactly compare molecular affinities among molecules with a fine distinction. Actually, differences in binding affinities among PAX6 mutants produced by ITC measurements were too small to be distinguished by other traditional studies such as EMSA. Interestingly, we found that *PAX6* mutation showed distinctive effects depending on target DNA sequences. For example, the p.Gly73Asp mutation showed no binding affinity for the promoter DNA sequence required for the expression of B-cell surface protein CD19 (see Supplementary Table S3), but mild effects on P6B (Table 2) and P6CON (see Supplementary Table S4) promoters for ζ-crystallin expression. We assume that the use of different target DNA sequence might lead to conflicting results for DNA-binding affinity compared to a previous study using EMSA. It has been reported that *PAX6* has consensus DNA sequences to be recognized. Based on PAX6 crystal structure, PAI and RED subdomains of PAX6 might interact with consensus “TTCACGC” and “TG” sequences, respectively. The linker loop between PAI and RED can specifically bind to consensus “ATGA” sequence^21^. Since P6B and P6CON have similar DNA sequence to be recognized by PAX6, effects caused by *PAX6* mutation could be quite similar to each other (Fig. 4B, and see Supplementary Fig. S2, and Table S4). However, the DNA sequence for CD19 A-ins is different from that of P6B or P6CON except six consensus sequences (CAC-G-GT). Since we have primarily focused on the analysis of PAX6-P6B/P6CON complex involved in the expression of ζ-crystallin, we believe that biochemical study of PAX6-P6B would be more reasonable to understand the function of *PAX6* mutation. Nevertheless, to gain further insight of structure and function relationship of PAX6 and its target promoter, complex structures of PAX6 bound to P6CON or CD19 A-ins may be needed.

In conclusion, we identified two novel mutations of *PAX6* gene in patients with iris hypoplasia and INS: c.214 G > T (p.Gly72Cys) and c.249_250delinsCGC (p.Val84Alafs*8) in exon 6. Using ITC measurement, we demonstrated that the mutation p.Val84Alafs*8 had no DNA binding affinity, while the p.Gly72Cys mutation showed a decreased binding affinity by approximately 1.4 times compared to the wild type-PAX6. We also found that there was no close relationship between DNA-binding affinity and phenotypic differences, suggesting that the DNA-binding affinity alone might not be sufficient to determine *PAX6*-related phenotypes, and other modifier genes or environmental factors might affect phenotypes of the *PAX6* gene.

## Methods

### Subjects and clinical assessment

Two patients were referred to the Neuro-ophthalmology Clinic of Pusan National University Yangsan Hospital for evaluation of infantile nystagmus syndrome (INS). Case 1 (P1-MSD) had a family history of INS consisting of four clinically affected and two unaffected members (Fig. 1A), and case 2 (P2-AJY) was a sporadic patient (Fig. 1C). They received detailed ophthalmic examinations, including measurement of best-corrected visual acuity (BCVA) using Snellen chart, ocular alignment, slit lamp examination of anterior and posterior segments, and a dilated fundus examination. High-resolution spectral-domain OCT (Visante OCT, Carl Zeiss Meditec, Dublin, CA, USA) was used to acquire tomograms of anterior and posterior segments. Detailed methods have been described previously^39,40^. In particular, the morphology of the anterior segment including iris anatomy and structure was carefully analyzed using anterior segment OCT. Foveal morphology obtained from posterior segment OCT was graded using the Foveal Hypoplasia Grading Scale^41^.

All experiments followed the tenets of the Declaration of Helsinki, and informed consent was obtained after the nature and possible consequences of this study had been explained to the participants. This study was approved by the Institutional Review Boards of Pusan National University Yangsan Hospital.

### Mutation analysis

Genomic DNA was extracted from the blood sample of each patient. Whole-exome sequencing (WES) was conducted using a SureSelect Focused Exome Kit (Agilent, Inc., USA). Detailed methods have been described previously^42^. Briefly, the qualified genomic DNA sample was randomly fragmented by Covaris followed by adapter ligation, purification, hybridization and PCR. Captured libraries were subjected to an Agilent 2100 Bioanalyzer to estimate the quality and loaded on to an Illumina HiSeq2500 (TheragenEtex Bio Institute, Suwon, Korea) according to the manufacturer’s recommendations. Raw image files were processed by HCS1.4.8 for base-calling with default parameters and sequences of each individual were generated as 100  bp paired-end reads.

Sequence reads were aligned to the human reference genome sequence (GRCh37.3, hg19) using the Burrows-Wheeler Aligner (BWA, version 0.7.12). PCR duplicate reads were marked and removed with Picard tools (version 1.92). Genome Analysis Toolkit (GATK, version 2.3–9) was used for indel realignment and base recalibration. Variation annotation and interpretation analysis were performed using SnpEff (version 4.2). To identify pathogenic mutations, 98 candidate genes associated with INS were selected based on literature reviews and Online Mendelian Inheritance in Man (OMIM) (see Supplementary Table S1)^43,44^. Variants causing non-synonymous amino acid changes, stop codons, insertions/deletions in coding regions, or changes to splice site sequences in exon/intron boundaries were identified. Common variants with minor allele frequency (MAF) > 0.01 that are present in dbSNP147, the Exome Aggregation Consortium (ExAC), the 1000 Genomes Project, and Korean Reference Genome Database (KRGDB: http://152.99.75.168/KRGDB/) were excluded. Variants screened with the above process were annotated for previously reported disease-causing variants using the Human Gene Mutation Database. Non-synonymous variants that were predicted as damaging by at least three of four prediction tools (SIFT, Polyphen2, LRT, MutationTaster), were considered as pathogenic mutations. These variants were confirmed by Sanger DNA sequencing, and screened in other family members and 150 Korean controls.

### Functional analysis of *PAX6* gene

#### Protein expression and purification

The gene encoding human PAX6 (residues 4–136) was amplified using PCR and inserted into a modified pETduet-1 vector, in which TEV protease cleavage site was included between His6-tag and PAX6 at the N-terminus. Protein production was carried out as previously described^21^, except for using Ni^2+^-affinity chromatography. His6 tag was removed by TEV protease during dialysis against buffer comprising 25 mM Tris⋅HCl, pH 7.5, 150 mM NaCl, and 5 mM β-mercaptoethanol (β-Me). Subsequently, protein solution was applied to a Superdex 200 16/60 column (GE Hralthcare) pre-equilibrated in 25 mM Hepes, pH 7.5, 150  mM NaCl, and 5  mM β-Me. Purified proteins were concentrated to ~10 mg/ml using a 10,000 NMWL Amicon Ultra centrifugal filter (Millipore) and flash-frozen in liquid nitrogen for storage. Our mutants (p.Gly72Cys and p.Val84Alafs*8) were generated by PCR-based mutagenesis, and purified with the same method as described above. Previously reported missense mutations within the linker region between two subdomains of the PD (p.Gly64Val, p.Gly72Ser, p.Gly73Asp, p.Ser74Gly and p.Pro76Arg), were also generated to compare DNA binding affinity. Among them, p.Gly64Val^23^, p.Ser74Gly^27^, and p.Pro76Arg^24^ have been reported to be associated with non-aniridia phenotype, whereas patients with p.Gly72Ser^25^ and p.Gly73Asp^26^ variant had an abnormal iris structure.

#### Isothermal titration calorimetry (ITC) measurement

DNA strands known to bind to PAX6 P6B (forward: 5′-AAGCATTTTCACGCATGAGTGCACAG; reverse: 5′-TTCTGTGCACTCATGCGTGAAAATGC), P6CON (forward: 5′-AAATTTTCACGCTTGAGTTCACAGCT; reverse: 5′-AGCTGTGAACTCAAGCGTGAAAATTT), and CD19 A-ins (forward: 5′-GAATGGGGCACTGAGGCGTGACCACCGC; reverse: 5′-GCGGTGGTCACGCCTCAGTGCCCCATTC) (see Supplementary Fig. S1) were chemically synthesized and purified using HPLC. Each strand was dissolved in buffer B and assembled into a duplex by mixing them and incubating at 95 °C for 5  min followed by cooling down to room temperature overnight. ITC measurements were performed using a Microcal iTC_200_ instrument (Malvern) as previously described^45^. All experiments were carried out in the buffer C at 25 °C. PAX6 and its mutants (20  μM) were placed into sample cells. DNA (200  μM) was injected using a syringe with 20 successive additions of 2  μl for 4  s with 1000  rpm stirring. Titration curves were fitted to one set of sites model using Malvern software supplied with the instrument.

## Supplementary information

Supplementary information.

## Data Availability

The datasets generated during the current study are available from the corresponding author on reasonable request.

## References

[1] Glaser T, Walton DS, Maas RL (1992). Genomic structure, evolutionary conservation and aniridia mutations in the human PAX6 gene. Nat. Genet..

[2] Wawrocka A, Krawczynski MR (2018). The genetics of aniridia - simple things become complicated. J. Appl. Genet..

[3] Ton CC (1991). Positional cloning and characterization of a paired box- and homeobox-containing gene from the aniridia region. Cell..

[4] Halder G, Callaerts P, Gehring WJ (1995). New perspectives on eye evolution. Curr. Opin. Genet. Dev..

[5] Simpson TI, Price DJ (2002). Pax6; a pleiotropic player in development. Bioessays..

[6] Treisman JE (2004). How to make an eye. Development..

[7] Lang D, Powell SK, Plummer RS, Young KP, Ruggeri BA (2007). PAX genes: roles in development, pathophysiology, and cancer. Biochem. Pharmacol..

[8] Kokotas H, Petersen M (2010). Clinical and molecular aspects of aniridia. Clin. Genet..

[9] Brown A, McKie M, van Heyningen V, Prosser J (1998). The human PAX6 mutation database. Nucleic Acids Res..

[10] The PAX6 Allelic Variant Database http://pax6.hgu.mrc.ac.uk/.

[11] Tzoulaki I, White IM, Hanson IM (2005). PAX6 mutations: genotype-phenotype correlations. BMC Genet..

[12] Hingorani M, Hanson I, van Heyningen V (2012). Aniridia. Eur. J. Hum. Genet..

[13] Robinson DO (2008). Genetic analysis of chromosome 11p13 and the PAX6 gene in a series of 125 cases referred with aniridia. Am. J. Med. Genet. A..

[14] Axton R (1997). The incidence of PAX6 mutation in patients with simple aniridia: an evaluation of mutation detection in 12 cases. J. Med. Genet..

[15] Zhuang J (2014). A novel de novo duplication mutation of PAX6 in a Chinese family with aniridia and other ocular abnormalities. Sci. Rep..

[16] Mirzayans F, Pearce WG, MacDonald IM, Walter MA (1995). Mutation of the PAX6 gene in patients with autosomal dominant keratitis. Am. J. Hum. Genet..

[17] Wang P (2012). PAX6 mutations identified in 4 of 35 families with microcornea. Invest. Ophthalmol. Vis. Sci..

[18] Azuma N (2003). Mutations of the PAX6 gene detected in patients with a variety of optic-nerve malformations. Am. J. Hum. Genet..

[19] Goolam S (2018). Familial congenital cataract, coloboma, and nystagmus phenotype with variable expression caused by mutation in PAX6 in a South African family. Mol. Vis..

[20] Hanson IM (1994). Mutations at the PAX6 locus are found in heterogeneous anterior segment malformations including Peters’ anomaly. Nat. Genet..

[21] Xu HE (1999). Crystal structure of the human Pax6 paired domain-DNA complex reveals specific roles for the linker region and carboxy-terminal subdomain in DNA binding. Genes. Dev..

[22] Tang HK, Chao LY, Saunders GF (1997). Functional analysis of paired box missense mutations in the PAX6 gene. Hum. Mol. Genet..

[23] Hanson I (1999). Missense mutations in the most ancient residues of the PAX6 paired domain underlie a spectrum of human congenital eye malformations. Hum. Mol. Genet..

[24] Thomas S (2014). Autosomal-dominant nystagmus, foveal hypoplasia and presenile cataract associated with a novel PAX6 mutation. Eur. J. Hum. Genet..

[25] Hingorani M, Williamson KA, Moore AT, van Heyningen V (2009). Detailed ophthalmologic evaluation of 43 individuals with PAX6 mutations. Invest. Ophthalmol. Vis. Sci..

[26] Chao LY, Mishra R, Strong LC, Saunders GF (2003). Missense mutations in the DNA-binding region and termination codon in PAX6. Hum. Mutat..

[27] Dansault A (2007). Three new PAX6 mutations including one causing an unusual ophthalmic phenotype associated with neurodevelopmental abnormalities. Mol. Vis..

[28] Chauhan BK, Yang Y, Cveklová K, Cvekl A (2007). Functional Properties of Natural Human PAX6 and PAX6(5a) Mutants. Invest. Ophthalmol. Vis. Sci..

[29] Czerny T, Schaffner G, Busslinger M (1993). DNA sequence recognition by Pax proteins: bipartite structure of the paired domain and its binding site. Genes. Dev..

[30] Glaser T (1994). PAX6 gene dosage effect in a family with congenital cataracts, aniridia, anophthalmia and central nervous system defects. Nat. Genet..

[31] Yokoi T (2016). Genotype-phenotype correlation of PAX6 gene mutations in aniridia. Hum. Genome Var..

[32] Azuma N, Hotta Y, Tanaka H, Yamada M (1998). Missense mutations in the PAX6 gene in aniridia. Invest. Ophthalmol. Vis. Sci..

[33] Pedersen HR (2019). The Cone Photoreceptor Mosaic in Aniridia: Within-Family Phenotype-Genotype Discordance. Ophthalmol. Retina..

[34] Cvekl A, Yang Y, Chauhan BK, Cveklova K (2004). Regulation of gene expression by Pax6 in ocular cells: a case of tissue-preferred expression of crystallins in lens. Int. J. Dev. Biol..

[35] Grocott T (2007). The MH1 domain of Smad3 interacts with Pax6 and represses autoregulation of the Pax6 P1 promoter. Nucleic Acids Res..

[36] Gauthier BR, Gosmain Y, Mamin A, Philippe J (2007). The beta-cell specific transcription facto Nkx6.1 inhibits glucagon gene transcription by interfering with Pax6. Biochem. J..

[37] Tabata H, Koinui A, Ogura A, Nishihara D, Yamamoto H (2018). A novel nuclear localization signal spans the linker of the two DNA-binding subdomains in the conserved paired domain of Pax6. Genes. Genet. Syst..

[38] Pierce MM, Raman CS, Nall BT (1999). Isothermal titration calorimetry of protein-protein interactions. Methods..

[39] Kim YC, Sung MS, Heo H, Park SW (2016). Anterior segment configuration as a predictive factor for refractive outcome after cataract surgery in patients with glaucoma. BMC Ophthalmol..

[40] Choi JH, Shin JH, Seo JH, Jung JH, Choi KD (2015). A start codon mutation of the FRMD7 gene in two Korean families with idiopathic infantile nystagmus. Sci. Rep..

[41] Thomas MG (2011). Structural grading of foveal hypoplasia using spectral-domain optical coherence tomography a predictor of visual acuity?. Ophthalmology..

[42] Choi KD (2017). Genetic Variants Associated with Episodic Ataxia in Korea. Sci. Rep..

[43] Rim JH (2017). Accuracy of Next-Generation Sequencing for Molecular Diagnosis in Patients With Infantile Nystagmus Syndrome. JAMA Ophthalmol..

[44] Thomas MG, Maconachie G, Sheth V, McLean RJ, Gottlob I (2017). Development and clinical utility of a novel diagnostic nystagmus gene panel using targeted next-generation sequencing. Eur. J. Hum. Genet..

[45] Jeong H (2017). Mechanistic insight into the nucleus-vacuole junction based on the Vac8p-Nvj1p crystal structure. Proc. Natl Acad. Sci. USA.

