# Supplementary material for: Impaired DNA-binding affinity of novel PAX6 mutations
Source: Sci Rep. 2020 Feb 20;10:3062. doi: 10.1038/s41598-020-60017-2 (PMC7046147; doi:10.1038/s41598-020-60017-2)
Supplement: Supplementary file 1 — Supplementary information. [file 41598_2020_60017_MOESM1_ESM.pdf]

Impaired DNA-binding affinity of novel *PAX6* mutations

Seowhang Lee, Seung-Han Lee, Hwan Heo, Eun Hye Oh, Jin-Hong Shin, Hyang-Sook Kim,
Jae-Ho Jung, Seo Young Choi, Kwang-Dong Choi, Hakbong Lee, Changwook Lee*, Jae-Hwan Choi*

*Corresponding authors: Changwook Lee (changwook@unist.ac.kr**),** Jae-Hwan Choi (rachelbolan@hanmail.net)

Supplementary Information:

Supplementary Figure S1: PAX6 binding DNA forward sequences used for ITC experiments.

Supplementary Figure S2: ITC measurement between wild-type PAX6 (residue: 4-136) and missense mutations (G64V, G72C, G72S, G73D, and S74G) according to its target DNA (P6CON and CD19 A-ins).

Supplementary Table S1: Target genes associated with infantile nystagmus syndrome

Supplementary TableS 2: Rare variants in infantile nystgmus syndrome-associated genes identified by targeted next-generation sequencing.

Supplementary Table S3: DNA (CD19 A-ins) binding affinities of *PAX6* mutations

Supplementary Table S4: DNA (P6CON) binding affinities of *PAX6* mutations

Pax6 binding DNA sequence

```
              5'                                                         3'
P6B           A A G C A T T T T C A C G C A T G A G T G C A C A G - - -
P6CON         - - A A A T T T T C A C G C T T G A G T T C A C A G C T -
CD19 A-ins    - G A A T G G G G C A C T G A G G C G T G A C C A C C G C
Consensus                       C A C - - - - G - G T - - - C A
```

Supplementary Figure S1. PAX6 binding DNA forward sequences (P6B, P6CON, and CD19 A-ins) used for isothermal titration calorimetry (ITC) experiments. According to PAX6 crystal structure, PAI and RED subdomains of PAX6 may interact with consensus “TTCACGC” and “TG” sequence, respectively. The linker loop between PAI and RED can specifically bind to consensus “ATGA” sequence. Both P6B and P6CON have similar DNA sequence to be recognized by PAX6, while DNA sequence for CD19 A-ins is different from that of P6B or P6CON except six consensus sequences. The consensus PAX6 recognition sequences (CAC-G-GT) are colored in red.

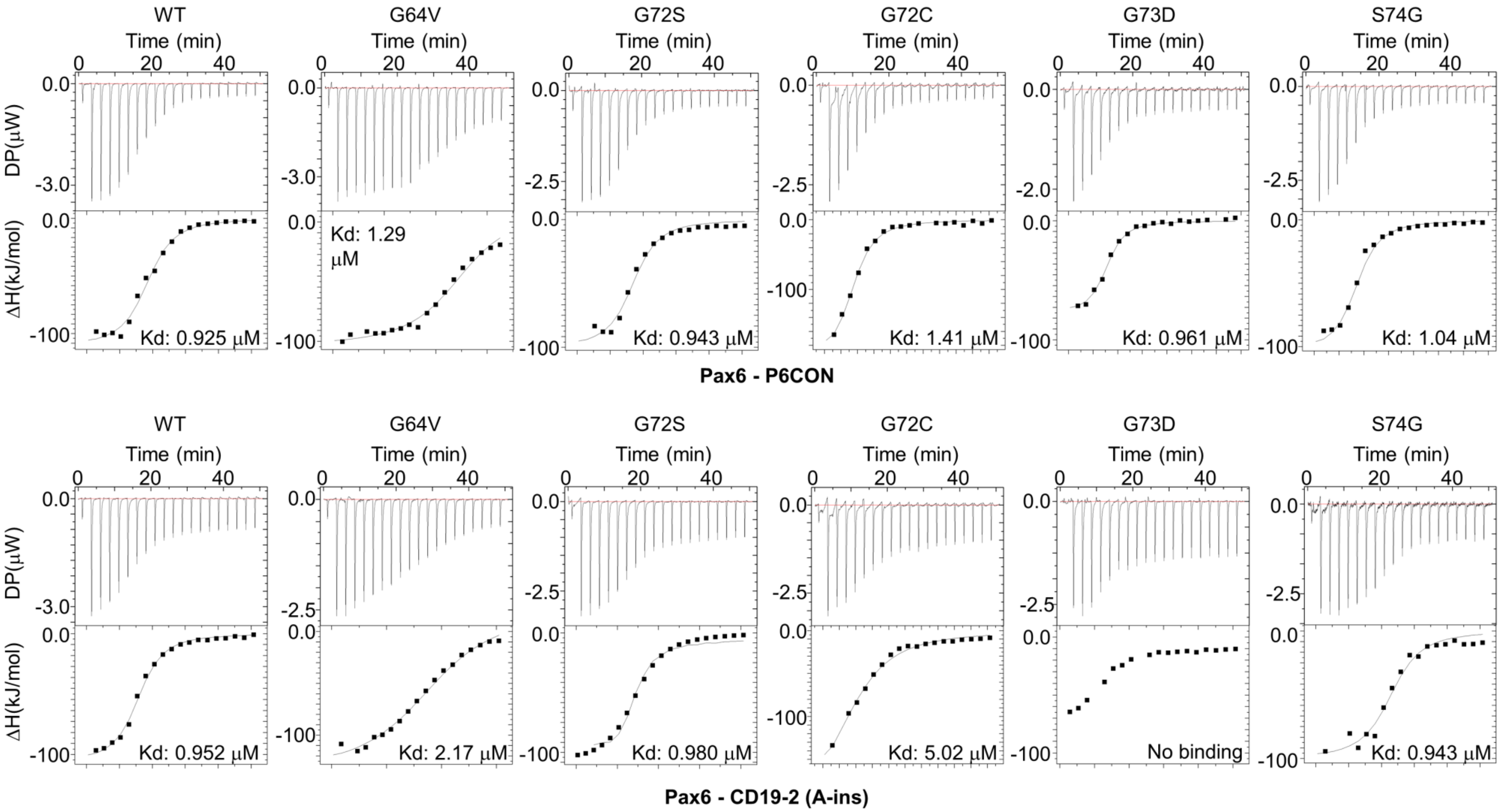

Supplementary Figure S2. ITC measurement between wild-type PAX6 (residue: 4-136) and missense mutations (G64V, G72C, G72S, G73D, and S74G) according to its target DNA (P6CON and CD19 A-ins). With regard to the G73D mutation that showed complete loss of DNA-binding affinity in a previous study using EMSA, the binding affinity of the mutant for P6CON (Kd = 0.961 μM) was unaffected compared to the WT (Kd = 0.925 μM) in our ITC measurement (upper panel). Whereas, the

binding affinity for CD19 A-ins was undetectable as shown in a previous study (low panel). With regard to the G64V mutation that exhibited an enhanced binding affinity to P6CON in a previous study using EMSA, our ITC measurement showed a decreased binding affinity for both P6CON and CD19 A-ins by 1.4 times (Kd = 1.29 μM) and 2.3 times (Kd = 2.17 μM), respectively.

Supplementary Table S1: Target genes associated with infantile nystagmus syndrome

Gene	MIM Gene ID
ABCA4	601691
ADAM9	602713
AHI1	608894
AIPL1	604392
ALMS1	606844
AP3B1	603401
ATF6	605537
ATXN7	607640
BEST1	607854
BLOC1S3	609762
BLOC1S6	604310
C10ORF11	614537
C8ORF37	614477
CABP4	608965
CACNA1F	300110
CACNA2D4	608171
CC2D2A	612013
CDH3	114021
CDHR1	609502
CEP290	610142
CERKL	608381
CHM	300390
CNGA3	600053
CNGB3	605080
CNNM4	607850
COL11A1	120280
COL2A1	120140
CRB1	604210
CRX	602225
CRYBA1	123610
CYP27A1	606530
DTNBP1	607145
ELOVL4	605512
FRMD7	300628
FSCN2	607643
FZD4	604579
GDF6	601147
GJC2	608803
GNAT2	139340
GPR143	300808
GUCA1A	600364
GUCA1B	602275
GUCY2D	600179
HPS1	604982
HPS3	606118
HPS4	606682
HPS5	607521
HPS6	607522
IMPDH1	146690
INPP5E	613037
IQCB1	609237
KCNJ13	603208
KCNV2	607604

LCA5	611408
LRAT	604863
LYST	606897
MITF	156845
MYO5A	160777
NDP	300658
NMNAT1	608700
NPHP1	607100
OCA2	611409
OPA1	605290
OPA3	606580
OTX2	600037
PAX2	167409
PAX6	607108
PDE6C	600827
PDE6H	601190
PITPNM3	608921
PROM1	604365
PRPH2	179605
RAB27A	603868
RAB28	612994
RAX2	610362
RD3	180040
RDH12	608830
RIMS1	606629
RP1L1	608581
RPE65	180069
RPGR	312610
RPGRIP1	605446
RPGRIP1L	610937
SEMA4A	607292
SLC24A5	609802
SLC38A8	615585
SLC45A2	606202
SOX10	602229
SPATA7	609868
TMEM126A	612988
TMEM216	613277
TMEM237	614423
TMEM67	609884
TULP1	602280
TYR	606933
TYRP1	115501
WDR19	608151
ZNF423	604557

Supplementary Table S2. Rare variants in infantile nystgmus syndrome-associated genes identified by targeted next-generation sequencing.

Gene	Transcript ID	Zygosity	mRNA	Protein	Variant effect	In silico prediction				ExAC	dbSNP	Phenotype (inheritance)
						SIFT	Polyphen	LRT	Mutation taster			
**Case 1 (P1-MSH)**												
PAX6	NM_000280.4	hetero	c.214G>T	p.Gly72Cys	missense	D	D	D	D	(-)	(-)	Aniridia (AD)<br>Foveal hypoplasia (AD)<br>Keratitis (AD)<br>Optic nerve hypoplasia (AD)
ALMS1	NM_015120.4	hetero	c.8192T>C	p.Val2731Ala	missense	D	B	N	N	0.00003	rs200859630	Alstrom syndrome (AR)
**Case 2 (P2-AJY)**												
PAX6	NM_000280.4	hetero	c.249_250 delinsCGC	p.Val84Alafs*8	deletion-insertion	(-)	(-)	(-)	(-)	(-)	(-)	Aniridia (AD)<br>Foveal hypoplasia (AD)<br>Keratitis (AD)<br>Optic nerve hypoplasia (AD)
ALMS1	NM_015120.4	hetero	c.5825G>A	p.Arg1941His	missense	T	B	N	N	0.0003	rs146669152	Alstrom syndrome (AR)
ABCA4	NM_000350.2	hetero	c.5593C>T	p.His1865Tyr	missense	T	B	N	D	0.0001	rs201707267	Cone-rod dystrophy 3 (AR)

SIFT- D (damaging), T (tolerated); Polyphen- D (probably damaging), P (possibly damaging), B (benign); LRT- D (deleterious), N (neutral); MutationTaster- D (disease_causing), N (polymorphism)

AD, autosomal dominant; AR, autosomal recessive

Supplementary Table S3. DNA (CD19 A-ins) binding affinities of *PAX6* mutations

Mutation	Iris anomaly	Kd (μM)	N	ΔH (kJ/mol)	TΔS (kJ/mol)	reference
WT	WT	0.952±0.078	0.778±0.012	-98±2.04	-214	(-)
p.Gly64Val	normal iris	2.170±0.162	0.974±0.017	-129±3.67	-326	[23]
p.Gly72Cys	iris hypoplasia	5.020±0.360	1.070±0.068	-179±14.8	-499	this study
p.Gly72Ser	iris hypoplasia	0.980±0.043	0.715±0.022	-110±5.03	-254	[25]
p.Gly73Asp	aniridia	no binding	(-)	(-)	(-)	[26]
p.Ser74Gly	normal iris	0.943±0.031	1.140±0.037	-100±5.17	-222	[27]

Kd is dissociation constant in equilibrium state; N, stoichiometry indicates the ratio of ligand-to-macromolecule binding; ΔH, enthalpy is indication of changes in hydrogen and van der Waals bonding; TΔS, entropy is indication of changes in hydrophobic interaction and conformational changes.

Supplementary Table S4. DNA (P6CON) binding affinities of *PAX6* mutations

Mutation	Iris anomaly	Kd (μM)	N	ΔH (kJ/mol)	TΔS (kJ/mol)	reference
WT	WT	0.925±0.047	0.911±0.020	-111±3.38	-258	(-)
p.Gly64Val	normal iris	1.290±0.078	1.250±0.016	-104±2.10	-236	[23]
p.Gly72Cys	iris hypoplasia	1.410±0.110	0.856±0.024	-202±7.74	-566	this study
p.Gly72Ser	iris hypoplasia	0.943±0.037	0.836±0.026	-100±4.80	-223	[25]
p.Gly73Asp	aniridia	0.961±0.048	0.721±0.014	-76.3±1.84	-141	[26]
p.Ser74Gly	normal iris	1.040±0.045	0.842±0.028	-104±4.77	-236	[27]

Kd is dissociation constant in equilibrium state; N, stoichiometry indicates the ratio of ligand-to-macromolecule binding; ΔH, enthalpy is indication of changes in hydrogen and van der Waals bonding; TΔS, entropy is indication of changes in hydrophobic interaction and conformational changes.
